# The crystal structure of 4′-{4-[(2,2,5,5-tetra­methyl-*N*-oxyl-3-pyrrolin-3-yl)ethyn­yl]phen­yl}-2,2′:6′,2′′-terpyridine

**DOI:** 10.1107/S2056989015012086

**Published:** 2015-06-30

**Authors:** Andreas Meyer, Jennifer Wiecek, Gregor Schnakenburg, Olav Schiemann

**Affiliations:** a University of Bonn, Institute of Physical and Theoretical Chemistry, Wegelerstrasse 12, 53115 Bonn, Germany; b University of Bonn, Institute of Inorganic Chemistry, Gerhard-Domagk-Strasse 1, 53121 Bonn, Germany

**Keywords:** crystal structure, terpyridine, nitrox­ide, nitrox­yl, C—H⋯π interactions, π–π interactions, C—H⋯O hydrogen bonding

## Abstract

The terpyridine group of the title compound, C_31_H_27_N_4_O, assumes an all-*transoid* conformation and is essentially planar with the dihedral angles between the mean planes of the central pyridine and the two outer rings amounting to 3.87 (5) and 1.98 (5)°. The pyrroline-*N*-oxyl group commonly seen in such nitroxyls is found in the title structure and the mean plane of the pyrroline ring subtends a dihedral angle of 88.44 (7)° to the mean plane of the central pyridine ring. The intra­molecular separation between the nitrogen atom of the central pyridine unit of the terpyridine group and the nitroxyl group is 14.120 (2) Å. In the crystal, the mol­ecules are arranged in layers stacked along [001]. Slipped face-to-face π–π inter­actions between the pyridine rings are observed along this direction with the shortest centroid–centroid distances amounting to 3.700 (1) and 3.781 (1) Å. Furthermore, edge-on C—H⋯π inter­actions between the phenyl­ene rings of neighbouring mol­ecules are observed along this direction. A two-dimensional C—H⋯O hydrogen-bonded network is formed within the (010) plane. The shortest O⋯O separation between neighbouring mol­ecules is 5.412 (3) Å.

## Abstract 2

The crystal structure of a nitroxide-substituted terpyridine mol­ecule is presented and discussed.

## Chemical context   

The title compound, (**1**), was synthesized as a ligand for 3*d* metal ions as part of a pulsed EPR study on metal–nitroxyl model systems. The mol­ecule contains a paramagnetic nitroxyl group and a terpyridine group. Nitroxyls have been the subject of magnetic studies in which exchange inter­actions have been detected (see, for example, Rajca *et al.*, 2006 ▸; Fritscher *et al.*, 2002 ▸). Furthermore, nitroxyls are used as spin labels for structural investigations of biological macromolecules (Reginsson & Schiemann, 2011 ▸). The structures of terpyridines have been investigated by Fallahpour *et al.* (1999 ▸), Eryazici *et al.* (2006 ▸), Bessel *et al.* (1992 ▸) and Grave *et al.* (2003 ▸) to name a few examples. The terpyridine moiety is known to form complexes with various metals. Numerous studies on metal complexes of terpyridine have been conducted, examples include those by Hogg & Wilkins (1962 ▸), Constable *et al.* (1999 ▸), Narr *et al.* (2002 ▸) and Folgado *et al.* (1990 ▸).
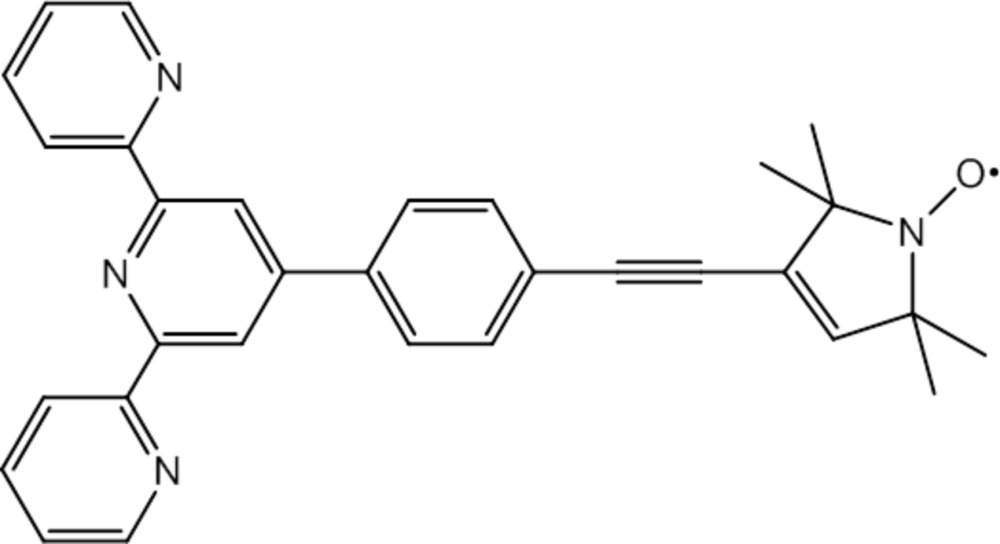


## Structural commentary   

**Figure 1:**
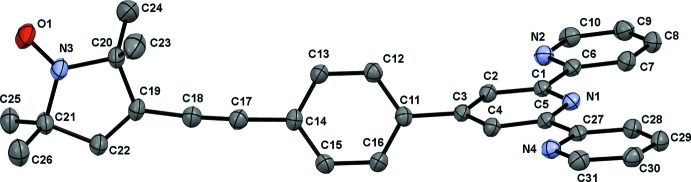
The mol­ecular structure of the title compound with displacement ellipsoids drawn at the 50% probability level. H atoms have been omitted for clarity.

The structure of the title compound (**1**) is shown in Fig. 1 ▸. The terpyridine group of (**1**) assumes an all-*transoid* conformation and is essentially planar with angles between the mean planes of the central pyridine (N1, C1–C5, r.m.s deviation from the mean plane = 0.006 Å) and the two outer rings amounting to 3.87 (5)° (N4, C27–C31, r.m.s. deviation from the mean plane = 0.003 Å) and 1.98 (5)° (N2, C6–C10, r.m.s deviation from the mean plane = 0.006 Å), respectively. The pyrroline-*N*-oxyl unit commonly found for such nitroxyls is seen in the structure and its mean plane (N3, C19–C22, r.m.s deviation from the mean plane = 0.006 Å) subtends a dihedral angle of 88.44 (7)° to the mean plane of the central pyridine ring (for similar structural motifs, see Margraf *et al.*, 2009 ▸ and Schuetz *et al.*, 2010 ▸). The subunits are linked by a 4-ethinylene­phenyl­ene group. The mean plane of the phenyl­ene group (C11–C16, r.m.s deviation from the mean plane < 0.001 Å) is tilted with respect to both the central pyridine ring [dihedral angle of 51.36 (5)°] and the pyrroline-*N*-oxyl [dihedral angle of 37.62 (7)°]. The angles C18—C17—C14 [177.35 (19)°] and C17—C18—C19 [175.64 (18)°] are slightly lower than the 180° expected for a strictly linear shape of the mol­ecular backbone. Two short intra­molecular hydrogen–nitro­gen distances are observed between the two *meta*-protons of the central pyridine subunit and the nitro­gen atoms of the external pyridine rings (Table 1 ▸). Murguly *et al.* (1999 ▸) propose weak intra­molecular hydrogen bonds for these atoms. The intra­molecular separation between the terpyridine group and the nitroxyl amounts to 14.120 (2) Å (measured between O1 and N1).

## Supra­molecular features   

**Figure 2:**
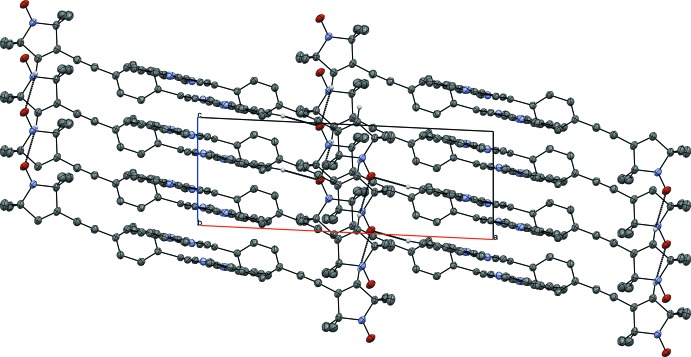
Crystal packing of the title compound viewed along the *b* axis. Weak C—H⋯O hydrogen bonds are shown as dashed lines

**Figure 3:**
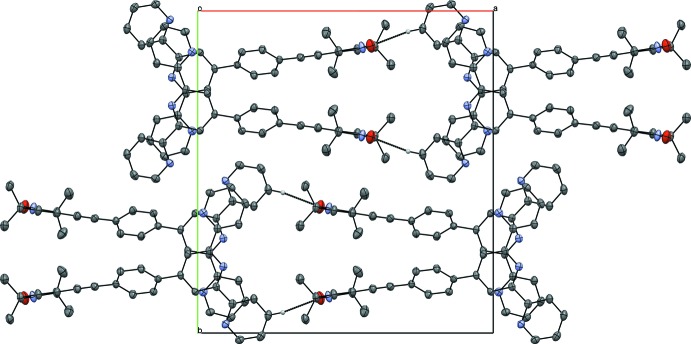
Crystal packing of the title compound viewed along the *c* axis.

**Figure 5:**
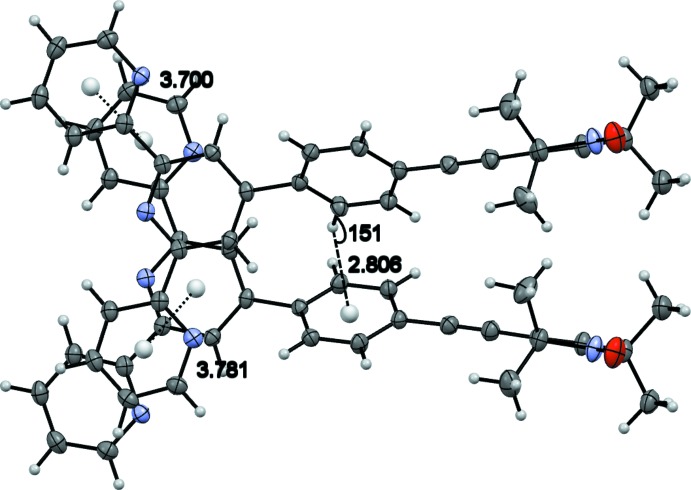
Closest distances between pyridine rings and edge-on C—H⋯π contact.

The packing within the crystal structure is shown in Figs. 2 ▸–4 ▸ ▸. The mol­ecules are stacked in layers along [001] (Fig. 2 ▸.) The oxygen atom of the nitroxyl group forms weak hydrogen bonds to the protons of the *para*-C—H group and the pyrroline C—H group of neighbouring mol­ecules (Table 1 ▸). These hydrogen bonds span a two-dimensional network within the (010) plane (Figs. 3 ▸ and 4 ▸). π–π inter­actions are observed along [001] between the terpyridine subunits of neighbouring mol­ecules (Figs. 3 ▸ and 5 ▸). These terpyridine subunits are arranged in a slipped face-to-face alignment (Janiak, 2000 ▸) with the shortest inter­molecular distances between the pyridine rings amounting to 3.700 (1) Å (measured from the centroid of N2, C6–C10 to the centroid of N4, C27–C31) and 3.781 (1) Å (centroid of N1, C1–C5 to the centroid of N4, C27–C31, see Fig. 5 ▸). Furthermore, the phenyl­ene rings of neighbouring mol­ecules show an edge-on C—H⋯π inter­action along the same axis (Table 1 ▸ and Fig. 5 ▸). The nitroxyl groups are arranged in an alternating manner pointing in opposite directions. The shortest oxygen–oxygen separation between neighbouring mol­ecules amounts to 5.412 (3) Å. The oxygen–oxygen distance is an important factor determining the strength of through space exchange inter­actions of nitroxyls (Rajca *et al.* 2006 ▸).

## Database survey   

The Cambridge Structural Database (CSD, Version 5.36; Groom & Allen, 2014 ▸) has been queried to find other terpyridine or 2,2,5,5-tetra­methyl-*N*-oxyl-3-pyrroline derivatives. The terpyridine query revealed 3473 entries in the CSD if metal complexes of terpyridine were included. For purely organic terpyridine compounds, the number of hits was reduced to 348. Only 33 results for 2,2,5,5-tetra­methyl-*N*-oxyl-3-pyrroline derivatives were found in the CSD. A combined query for structures which include both terpyridine and 2,2,5,5-tetra­methyl-*N*-oxyl-3-pyrroline derivatives did not result in any hit. However, the authors are aware of at least one published crystal structure of a compound which contains both structural motifs (Ackermann *et al.*, 2015 ▸).

## Synthesis and crystallization   

**Figure 6:**
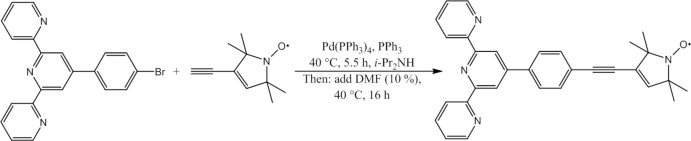
Scheme illustrating the synthesis of (**1**).

The title compound (**1**) is formed from 3-ethinyl-2,2,5,5-tetra­methyl-3-pyrroline-*N*-oxyl and 4′-(4-bromo­phen­yl)-2,2′:6′,2′′-terpyridine using a Sonogashira–Hagihara cross-coupling reaction, as shown in Fig. 6 ▸. 222 mg (0.57 mmol) of 4′-(4-bromo­phen­yl)-2,2′:6′,2′′-terpyridine, 100 mg (0.61 mmol) of 3-ethinyl-2,2,5,5-tetra­methyl-3-pyrroline-*N*-oxyl, 20 mg (0.076 mmol) of PPh_3_ and 40 mg (0.035 mmol) of Pd(PPh_3_)_4_ were dissolved in 17 ml of *i*-Pr_2_NH and stirred at 313 K, yielding a yellow solution which turned orange over the course of 5 min. Additionally, an orange precipitate was formed simultaneously. After 5.5 h, 2 ml of di­methyl­formamide were added to the orange suspension. The stirring at 313 K was continued for 16 h, after which time the solvents were removed under reduced pressure. The orange residues were suspended in a mixture of di­chloro­methane and cyclo­hexane (1:2) and subsequently subjected to column chromatography using aluminum oxide as stationary phase. A mixture of di­chloro­methane and cyclo­hexane was used as eluent. The volumetric ratio of both solvents was changed stepwise during the purification (from 1:8 to 8:1). The desired product was obtained in a yellow fraction and could be isolated by removing the eluents under reduced pressure (yield 80%). The crystallization of (**1**) was achieved by slow evaporation of a solution of (**1**) in a 1:1 mixture of aceto­nitrile and di­chloro­methane. 4′-(4-Bromo­phen­yl)-2,2′:6′,2′′-terpyridine was purchased from TCI Europe. 3-Ethinyl-2,2,5,5-tetra­methyl-3-pyrroline-*N*-oxyl was synthesized as described by Schiemann *et al.* (2007 ▸).

## Refinement   

Crystal data, data collection and structure refinement details are summarized in Table 2 ▸. All H atoms were fixed geometrically and allowed to ride on their parent C atoms, with 0.98 Å with *U*_iso_(H) = 1.5*U*_eq_(C) for methyl H atoms and C—H = 0.95 Å and *U*_iso_(H) = 1.2*U*_eq_(C) for all other H atoms.

## Supplementary Material

Crystal structure: contains datablock(s) I. DOI: 10.1107/S2056989015012086/lh5769sup1.cif

Structure factors: contains datablock(s) I. DOI: 10.1107/S2056989015012086/lh5769Isup2.hkl

Supporting information file. DOI: 10.1107/S2056989015012086/lh5769Isup3.cdx

CCDC reference: 1408457

Additional supporting information:  crystallographic information; 3D view; checkCIF report

## Figures and Tables

**Figure 4:**
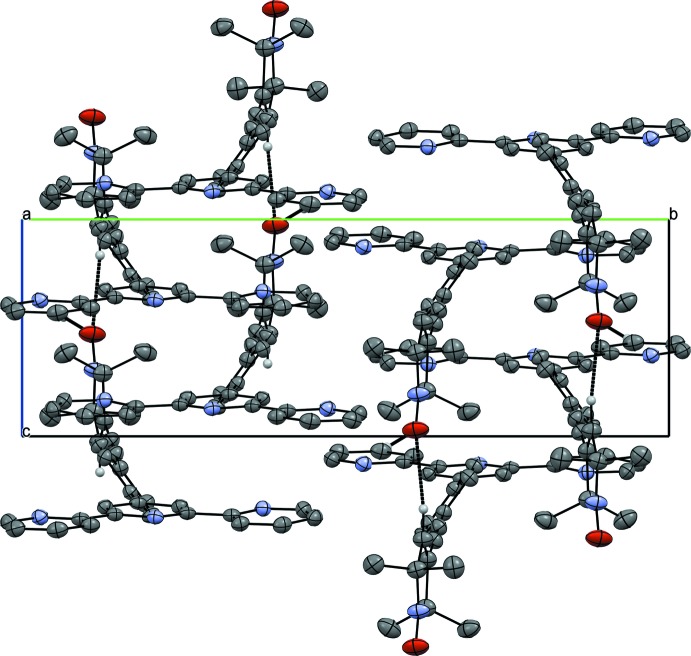
Crystal packing of the title compound viewed along the *a* axis.

**Table 1:** Hydrogen-bond geometry (, ) *Cg* is the centroid of the C11C16 ring.

*D*H*A*	*D*H	H*A*	*D* *A*	*D*H*A*
C2H2N2	0.95	2.50	2.815(2)	99
C4H4N4	0.95	2.46	2.778(2)	100
C8H8O1^i^	0.95	2.59	3.529(2)	170
C16H16*Cg* ^ii^	0.95	2.81	3.669(2)	151
C22H22O1^iii^	0.95	2.55	3.485(2)	170

Symmetry codes: (i) 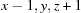; (ii) 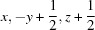; (iii) 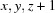.

**Table 2:** Experimental details

Crystal data	
Chemical formula	C_31_H_27_N_4_O
*M*_r_	471.56
Crystal system, space group	Monoclinic, *P*2_1_/*c*
Temperature (K)	123
*a*, *b*, *c* ()	18.5666(8), 20.2009(9), 6.7749(2)
()	92.743(3)
*V* (^3^)	2538.10(17)
*Z*	4
Radiation type	Mo *K*
(mm^1^)	0.08
Crystal size (mm)	0.34 0.12 0.08

Data collection	
Diffractometer	Nonius KappaCCD
Absorption correction	Multi-scan (Blessing, 1995 ▸)
*T*_min_, *T*_max_	0.883, 1.078
No. of measured, independent and observed [*I* > 2(*I*)] reflections	35758, 6691, 3221
*R*_int_	0.118
(sin /)_max_ (^1^)	0.685

Refinement	
*R*[*F* ^2^ > 2(*F* ^2^)], *wR*(*F* ^2^), *S*	0.049, 0.122, 0.89
No. of reflections	6691
No. of parameters	329
H-atom treatment	H-atom parameters constrained
_max_, _min_ (e ^3^)	0.19, 0.23

Computer programs: *DENZO* and *SCALEPACK* (Otwinowski Minor, 1997 ▸), *SHELXS97* (Sheldrick, 2008 ▸), *SHELXL97* (Sheldrick, 2015 ▸) and *OLEX2* (Dolomanov *et al.*, 2009 ▸).

## supplementary crystallographic information

### Crystal data

**Table:**

C_31_H_27_N_4_O	*F*(000) = 996
*M**_r_* = 471.56	*D*_x_ = 1.234 Mg m^−^^3^
Monoclinic, *P*2_1_/*c*	Mo *K*α radiation, λ = 0.71073 Å
*a* = 18.5666 (8) Å	Cell parameters from 9616 reflections
*b* = 20.2009 (9) Å	θ = 1.0–29.1°
*c* = 6.7749 (2) Å	µ = 0.08 mm^−^^1^
β = 92.743 (3)°	*T* = 123 K
*V* = 2538.10 (17) Å^3^	Needle, clear yellow
*Z* = 4	0.34 × 0.12 × 0.08 mm

### Data collection

**Table:**

Nonius KappaCCD diffractometer	6691 independent reflections
Radiation source: sealed tube	3221 reflections with *I* > 2σ(*I*)
Graphite monochromator	*R*_int_ = 0.118
Detector resolution: 8 pixels mm^-1^	θ_max_ = 29.2°, θ_min_ = 3.0°
fine slicing ω and φ scans	*h* = −25→24
Absorption correction: multi-scan (Blessing, 1995)	*k* = −24→27
*T*_min_ = 0.883, *T*_max_ = 1.078	*l* = −9→6
35758 measured reflections	

### Refinement

**Table:**

Refinement on *F*^2^	0 restraints
Least-squares matrix: full	Hydrogen site location: inferred from neighbouring sites
*R*[*F*^2^ > 2σ(*F*^2^)] = 0.049	H-atom parameters constrained
*wR*(*F*^2^) = 0.122	*w* = 1/[σ^2^(*F*_o_^2^) + (0.052*P*)^2^] where *P* = (*F*_o_^2^ + 2*F*_c_^2^)/3
*S* = 0.89	(Δ/σ)_max_ < 0.001
6691 reflections	Δρ_max_ = 0.19 e Å^−^^3^
329 parameters	Δρ_min_ = −0.23 e Å^−^^3^

### Special details

**Table:**

Geometry. All e.s.d.'s (except the e.s.d. in the dihedral angle between two l.s. planes) are estimated using the full covariance matrix. The cell e.s.d.'s are taken into account individually in the estimation of e.s.d.'s in distances, angles and torsion angles; correlations between e.s.d.'s in cell parameters are only used when they are defined by crystal symmetry. An approximate (isotropic) treatment of cell e.s.d.'s is used for estimating e.s.d.'s involving l.s. planes.

### Fractional atomic coordinates and isotropic or equivalent isotropic displacement parameters (Å^2^)

**Table:**

	*x*	*y*	*z*	*U*_iso_*/*U*_eq_	
O1	0.58556 (7)	0.39166 (8)	0.02921 (17)	0.0449 (4)	
N1	−0.08706 (7)	0.29387 (7)	0.87301 (18)	0.0235 (3)	
N2	−0.09424 (7)	0.47223 (8)	0.87295 (19)	0.0264 (4)	
N3	0.55647 (8)	0.38695 (8)	0.1947 (2)	0.0332 (4)	
N4	−0.01906 (7)	0.12743 (7)	0.83306 (19)	0.0262 (3)	
C1	−0.06315 (9)	0.35653 (9)	0.8599 (2)	0.0221 (4)	
C2	0.00840 (9)	0.37160 (9)	0.8258 (2)	0.0228 (4)	
H2	0.0234	0.4164	0.8158	0.027*	
C3	0.05751 (9)	0.32063 (9)	0.8067 (2)	0.0224 (4)	
C4	0.03323 (9)	0.25616 (9)	0.8239 (2)	0.0236 (4)	
H4	0.0658	0.2202	0.8149	0.028*	
C5	−0.03946 (9)	0.24445 (9)	0.8545 (2)	0.0223 (4)	
C6	−0.11788 (9)	0.40962 (9)	0.8820 (2)	0.0244 (4)	
C7	−0.19006 (9)	0.39411 (9)	0.9094 (2)	0.0280 (4)	
H7	−0.2054	0.3493	0.9130	0.034*	
C8	−0.23880 (10)	0.44516 (10)	0.9310 (2)	0.0316 (5)	
H8	−0.2882	0.4359	0.9494	0.038*	
C9	−0.21466 (10)	0.50989 (10)	0.9254 (2)	0.0319 (5)	
H9	−0.2467	0.5459	0.9426	0.038*	
C10	−0.14220 (10)	0.52080 (9)	0.8941 (2)	0.0292 (4)	
H10	−0.1258	0.5653	0.8872	0.035*	
C11	0.13389 (9)	0.33241 (9)	0.7586 (2)	0.0228 (4)	
C12	0.14938 (9)	0.37194 (9)	0.5973 (2)	0.0260 (4)	
H12	0.1113	0.3937	0.5245	0.031*	
C13	0.21936 (9)	0.37982 (9)	0.5426 (2)	0.0273 (4)	
H13	0.2290	0.4069	0.4323	0.033*	
C14	0.27657 (9)	0.34827 (9)	0.6476 (2)	0.0244 (4)	
C15	0.26114 (9)	0.30872 (9)	0.8088 (2)	0.0273 (4)	
H15	0.2992	0.2870	0.8818	0.033*	
C16	0.19076 (9)	0.30096 (9)	0.8632 (2)	0.0273 (4)	
H16	0.1810	0.2738	0.9733	0.033*	
C17	0.34837 (10)	0.35565 (9)	0.5825 (2)	0.0275 (4)	
C18	0.40740 (9)	0.36297 (9)	0.5209 (2)	0.0294 (4)	
C19	0.47510 (9)	0.37261 (9)	0.4332 (2)	0.0267 (4)	
C20	0.47826 (9)	0.37532 (10)	0.2098 (2)	0.0294 (4)	
C21	0.59915 (9)	0.38953 (10)	0.3860 (2)	0.0305 (4)	
C22	0.53960 (9)	0.38011 (10)	0.5256 (3)	0.0308 (4)	
H22	0.5471	0.3796	0.6653	0.037*	
C23	0.45763 (11)	0.30952 (11)	0.1131 (3)	0.0445 (6)	
H23A	0.4643	0.3122	−0.0293	0.067*	
H23B	0.4070	0.2997	0.1357	0.067*	
H23C	0.4883	0.2743	0.1706	0.067*	
C24	0.43549 (11)	0.43246 (11)	0.1165 (3)	0.0444 (6)	
H24A	0.4511	0.4741	0.1791	0.067*	
H24B	0.3840	0.4257	0.1354	0.067*	
H24C	0.4438	0.4344	−0.0252	0.067*	
C25	0.63565 (10)	0.45658 (10)	0.4093 (3)	0.0374 (5)	
H25A	0.6686	0.4631	0.3023	0.056*	
H25B	0.6628	0.4584	0.5368	0.056*	
H25C	0.5990	0.4915	0.4039	0.056*	
C26	0.65362 (10)	0.33278 (11)	0.3968 (3)	0.0421 (5)	
H26A	0.6280	0.2904	0.3849	0.063*	
H26B	0.6812	0.3344	0.5236	0.063*	
H26C	0.6866	0.3371	0.2887	0.063*	
C27	−0.06712 (9)	0.17596 (9)	0.8639 (2)	0.0230 (4)	
C28	−0.13860 (9)	0.16253 (9)	0.9022 (2)	0.0268 (4)	
H28	−0.1714	0.1976	0.9238	0.032*	
C29	−0.16099 (10)	0.09759 (9)	0.9083 (2)	0.0295 (4)	
H29	−0.2095	0.0873	0.9348	0.035*	
C30	−0.11244 (10)	0.04767 (9)	0.8756 (2)	0.0295 (4)	
H30	−0.1268	0.0025	0.8789	0.035*	
C31	−0.04244 (10)	0.06489 (9)	0.8381 (2)	0.0290 (4)	
H31	−0.0091	0.0304	0.8145	0.035*	

### Atomic displacement parameters (Å^2^)

**Table:**

	*U*^11^	*U*^22^	*U*^33^	*U*^12^	*U*^13^	*U*^23^
O1	0.0353 (8)	0.0713 (12)	0.0294 (7)	−0.0087 (7)	0.0138 (6)	−0.0024 (7)
N1	0.0240 (8)	0.0271 (9)	0.0196 (7)	0.0008 (7)	0.0026 (6)	−0.0010 (6)
N2	0.0257 (9)	0.0283 (10)	0.0253 (7)	0.0025 (7)	0.0029 (6)	0.0004 (6)
N3	0.0245 (9)	0.0507 (12)	0.0250 (8)	−0.0073 (8)	0.0075 (6)	−0.0021 (7)
N4	0.0273 (8)	0.0282 (10)	0.0231 (7)	−0.0011 (7)	0.0018 (6)	−0.0003 (6)
C1	0.0209 (10)	0.0277 (11)	0.0179 (8)	−0.0020 (8)	0.0016 (6)	0.0002 (7)
C2	0.0224 (9)	0.0242 (10)	0.0219 (8)	−0.0020 (8)	0.0033 (6)	0.0002 (7)
C3	0.0188 (9)	0.0301 (11)	0.0183 (8)	−0.0022 (8)	0.0025 (6)	0.0000 (7)
C4	0.0218 (10)	0.0274 (11)	0.0221 (8)	0.0017 (8)	0.0043 (7)	0.0012 (7)
C5	0.0218 (9)	0.0284 (11)	0.0171 (7)	−0.0018 (8)	0.0032 (6)	0.0008 (7)
C6	0.0234 (10)	0.0314 (11)	0.0187 (8)	0.0007 (8)	0.0029 (7)	0.0003 (7)
C7	0.0239 (10)	0.0345 (12)	0.0259 (9)	−0.0004 (9)	0.0041 (7)	0.0001 (8)
C8	0.0225 (10)	0.0445 (14)	0.0282 (9)	0.0035 (9)	0.0055 (7)	0.0023 (8)
C9	0.0287 (11)	0.0387 (13)	0.0285 (9)	0.0104 (9)	0.0040 (7)	0.0033 (8)
C10	0.0329 (11)	0.0287 (11)	0.0261 (9)	0.0028 (9)	0.0018 (7)	0.0013 (8)
C11	0.0207 (9)	0.0234 (10)	0.0243 (8)	−0.0003 (8)	0.0026 (7)	−0.0023 (7)
C12	0.0234 (10)	0.0253 (11)	0.0293 (9)	0.0017 (8)	0.0020 (7)	0.0017 (7)
C13	0.0241 (10)	0.0316 (11)	0.0267 (9)	−0.0001 (8)	0.0053 (7)	0.0062 (8)
C14	0.0204 (9)	0.0255 (11)	0.0277 (9)	−0.0011 (8)	0.0060 (7)	−0.0007 (7)
C15	0.0213 (10)	0.0310 (11)	0.0297 (9)	0.0007 (8)	0.0011 (7)	0.0040 (8)
C16	0.0244 (10)	0.0314 (11)	0.0263 (9)	−0.0024 (8)	0.0035 (7)	0.0049 (8)
C17	0.0261 (11)	0.0279 (11)	0.0288 (9)	−0.0010 (8)	0.0035 (8)	0.0025 (7)
C18	0.0257 (11)	0.0320 (12)	0.0306 (9)	−0.0015 (9)	0.0031 (8)	0.0027 (8)
C19	0.0216 (10)	0.0292 (11)	0.0302 (9)	−0.0006 (8)	0.0085 (7)	0.0005 (8)
C20	0.0206 (10)	0.0379 (12)	0.0300 (9)	−0.0058 (9)	0.0042 (7)	0.0007 (8)
C21	0.0208 (10)	0.0406 (13)	0.0304 (9)	−0.0031 (9)	0.0039 (7)	−0.0028 (8)
C22	0.0236 (10)	0.0409 (13)	0.0281 (9)	−0.0031 (9)	0.0047 (7)	−0.0003 (8)
C23	0.0447 (13)	0.0543 (15)	0.0347 (11)	−0.0176 (11)	0.0054 (9)	−0.0079 (10)
C24	0.0350 (12)	0.0571 (16)	0.0414 (11)	0.0052 (11)	0.0050 (9)	0.0139 (10)
C25	0.0267 (11)	0.0446 (14)	0.0416 (11)	−0.0060 (9)	0.0078 (8)	−0.0031 (9)
C26	0.0297 (11)	0.0444 (14)	0.0526 (13)	0.0000 (10)	0.0070 (9)	0.0015 (10)
C27	0.0229 (10)	0.0299 (11)	0.0164 (8)	0.0001 (8)	0.0006 (6)	0.0003 (7)
C28	0.0234 (10)	0.0322 (12)	0.0248 (9)	−0.0010 (9)	0.0023 (7)	0.0017 (8)
C29	0.0245 (10)	0.0365 (12)	0.0276 (9)	−0.0069 (9)	0.0024 (7)	0.0021 (8)
C30	0.0336 (11)	0.0279 (11)	0.0270 (9)	−0.0070 (9)	0.0007 (7)	0.0022 (8)
C31	0.0327 (11)	0.0270 (11)	0.0273 (9)	−0.0020 (9)	0.0011 (7)	−0.0019 (8)

### Geometric parameters (Å, º)

**Table:**

O1—N3	1.2712 (17)	C15—H15	0.9500
N1—C1	1.346 (2)	C15—C16	1.383 (2)
N1—C5	1.343 (2)	C16—H16	0.9500
N2—C6	1.341 (2)	C17—C18	1.200 (2)
N2—C10	1.337 (2)	C18—C19	1.429 (2)
N3—C20	1.479 (2)	C19—C20	1.519 (2)
N3—C21	1.487 (2)	C19—C22	1.333 (2)
N4—C27	1.349 (2)	C20—C23	1.523 (3)
N4—C31	1.337 (2)	C20—C24	1.521 (3)
C1—C2	1.393 (2)	C21—C22	1.501 (2)
C1—C6	1.490 (2)	C21—C25	1.519 (3)
C2—H2	0.9500	C21—C26	1.528 (3)
C2—C3	1.386 (2)	C22—H22	0.9500
C3—C4	1.385 (2)	C23—H23A	0.9800
C3—C11	1.489 (2)	C23—H23B	0.9800
C4—H4	0.9500	C23—H23C	0.9800
C4—C5	1.396 (2)	C24—H24A	0.9800
C5—C27	1.478 (2)	C24—H24B	0.9800
C6—C7	1.397 (2)	C24—H24C	0.9800
C7—H7	0.9500	C25—H25A	0.9800
C7—C8	1.384 (2)	C25—H25B	0.9800
C8—H8	0.9500	C25—H25C	0.9800
C8—C9	1.383 (3)	C26—H26A	0.9800
C9—H9	0.9500	C26—H26B	0.9800
C9—C10	1.389 (2)	C26—H26C	0.9800
C10—H10	0.9500	C27—C28	1.391 (2)
C11—C12	1.395 (2)	C28—H28	0.9500
C11—C16	1.396 (2)	C28—C29	1.377 (2)
C12—H12	0.9500	C29—H29	0.9500
C12—C13	1.377 (2)	C29—C30	1.377 (3)
C13—H13	0.9500	C30—H30	0.9500
C13—C14	1.403 (2)	C30—C31	1.381 (2)
C14—C15	1.394 (2)	C31—H31	0.9500
C14—C17	1.432 (2)		
			
C5—N1—C1	118.19 (14)	C22—C19—C18	127.46 (16)
C10—N2—C6	117.76 (15)	C22—C19—C20	112.80 (15)
O1—N3—C20	122.18 (13)	N3—C20—C19	99.16 (13)
O1—N3—C21	122.33 (13)	N3—C20—C23	109.66 (15)
C20—N3—C21	115.43 (12)	N3—C20—C24	110.21 (15)
C31—N4—C27	117.70 (15)	C19—C20—C23	112.11 (16)
N1—C1—C2	122.47 (16)	C19—C20—C24	113.40 (16)
N1—C1—C6	116.22 (15)	C24—C20—C23	111.60 (16)
C2—C1—C6	121.31 (16)	N3—C21—C22	99.62 (13)
C1—C2—H2	120.3	N3—C21—C25	109.78 (15)
C3—C2—C1	119.35 (16)	N3—C21—C26	109.83 (15)
C3—C2—H2	120.3	C22—C21—C25	112.68 (15)
C2—C3—C11	122.65 (16)	C22—C21—C26	112.36 (16)
C4—C3—C2	118.22 (15)	C25—C21—C26	111.89 (15)
C4—C3—C11	119.06 (15)	C19—C22—C21	112.98 (15)
C3—C4—H4	120.2	C19—C22—H22	123.5
C3—C4—C5	119.52 (17)	C21—C22—H22	123.5
C5—C4—H4	120.2	C20—C23—H23A	109.5
N1—C5—C4	122.23 (16)	C20—C23—H23B	109.5
N1—C5—C27	117.40 (15)	C20—C23—H23C	109.5
C4—C5—C27	120.36 (16)	H23A—C23—H23B	109.5
N2—C6—C1	116.62 (15)	H23A—C23—H23C	109.5
N2—C6—C7	122.39 (16)	H23B—C23—H23C	109.5
C7—C6—C1	120.99 (17)	C20—C24—H24A	109.5
C6—C7—H7	120.6	C20—C24—H24B	109.5
C8—C7—C6	118.88 (18)	C20—C24—H24C	109.5
C8—C7—H7	120.6	H24A—C24—H24B	109.5
C7—C8—H8	120.4	H24A—C24—H24C	109.5
C9—C8—C7	119.14 (17)	H24B—C24—H24C	109.5
C9—C8—H8	120.4	C21—C25—H25A	109.5
C8—C9—H9	120.9	C21—C25—H25B	109.5
C8—C9—C10	118.13 (17)	C21—C25—H25C	109.5
C10—C9—H9	120.9	H25A—C25—H25B	109.5
N2—C10—C9	123.68 (18)	H25A—C25—H25C	109.5
N2—C10—H10	118.2	H25B—C25—H25C	109.5
C9—C10—H10	118.2	C21—C26—H26A	109.5
C12—C11—C3	119.82 (15)	C21—C26—H26B	109.5
C12—C11—C16	118.62 (15)	C21—C26—H26C	109.5
C16—C11—C3	121.41 (15)	H26A—C26—H26B	109.5
C11—C12—H12	119.7	H26A—C26—H26C	109.5
C13—C12—C11	120.64 (16)	H26B—C26—H26C	109.5
C13—C12—H12	119.7	N4—C27—C5	116.10 (15)
C12—C13—H13	119.6	N4—C27—C28	122.10 (17)
C12—C13—C14	120.79 (16)	C28—C27—C5	121.80 (16)
C14—C13—H13	119.6	C27—C28—H28	120.5
C13—C14—C17	119.32 (15)	C29—C28—C27	118.90 (17)
C15—C14—C13	118.61 (15)	C29—C28—H28	120.5
C15—C14—C17	122.03 (16)	C28—C29—H29	120.3
C14—C15—H15	119.8	C28—C29—C30	119.45 (17)
C16—C15—C14	120.38 (16)	C30—C29—H29	120.3
C16—C15—H15	119.8	C29—C30—H30	120.9
C11—C16—H16	119.5	C29—C30—C31	118.29 (18)
C15—C16—C11	120.96 (16)	C31—C30—H30	120.9
C15—C16—H16	119.5	N4—C31—C30	123.55 (17)
C18—C17—C14	177.35 (19)	N4—C31—H31	118.2
C17—C18—C19	175.64 (18)	C30—C31—H31	118.2
C18—C19—C20	119.74 (15)		
			
O1—N3—C20—C19	178.70 (16)	C6—C7—C8—C9	−0.2 (2)
O1—N3—C20—C23	61.1 (2)	C7—C8—C9—C10	1.3 (2)
O1—N3—C20—C24	−62.1 (2)	C8—C9—C10—N2	−1.4 (2)
O1—N3—C21—C22	−178.63 (17)	C10—N2—C6—C1	−179.46 (13)
O1—N3—C21—C25	62.9 (2)	C10—N2—C6—C7	1.0 (2)
O1—N3—C21—C26	−60.5 (2)	C11—C3—C4—C5	−175.31 (13)
N1—C1—C2—C3	−0.7 (2)	C11—C12—C13—C14	0.0 (3)
N1—C1—C6—N2	178.75 (13)	C12—C11—C16—C15	0.1 (3)
N1—C1—C6—C7	−1.7 (2)	C12—C13—C14—C15	0.0 (3)
N1—C5—C27—N4	176.14 (13)	C12—C13—C14—C17	177.81 (16)
N1—C5—C27—C28	−3.8 (2)	C13—C14—C15—C16	0.0 (3)
N2—C6—C7—C8	−1.0 (2)	C14—C15—C16—C11	−0.1 (3)
N3—C21—C22—C19	0.7 (2)	C16—C11—C12—C13	−0.1 (3)
N4—C27—C28—C29	−0.1 (2)	C17—C14—C15—C16	−177.74 (17)
C1—N1—C5—C4	0.5 (2)	C18—C19—C20—N3	178.91 (16)
C1—N1—C5—C27	−178.37 (13)	C18—C19—C20—C23	−65.4 (2)
C1—C2—C3—C4	−0.5 (2)	C18—C19—C20—C24	62.1 (2)
C1—C2—C3—C11	176.34 (14)	C18—C19—C22—C21	−179.71 (19)
C1—C6—C7—C8	179.43 (14)	C20—N3—C21—C22	−1.4 (2)
C2—C1—C6—N2	−1.8 (2)	C20—N3—C21—C25	−119.89 (17)
C2—C1—C6—C7	177.78 (15)	C20—N3—C21—C26	116.68 (17)
C2—C3—C4—C5	1.6 (2)	C20—C19—C22—C21	0.1 (2)
C2—C3—C11—C12	−51.0 (2)	C21—N3—C20—C19	1.5 (2)
C2—C3—C11—C16	133.51 (18)	C21—N3—C20—C23	−116.05 (17)
C3—C4—C5—N1	−1.7 (2)	C21—N3—C20—C24	120.71 (17)
C3—C4—C5—C27	177.14 (13)	C22—C19—C20—N3	−1.0 (2)
C3—C11—C12—C13	−175.65 (16)	C22—C19—C20—C23	114.73 (18)
C3—C11—C16—C15	175.58 (16)	C22—C19—C20—C24	−117.78 (18)
C4—C3—C11—C12	125.74 (17)	C25—C21—C22—C19	117.02 (18)
C4—C3—C11—C16	−49.7 (2)	C26—C21—C22—C19	−115.47 (18)
C4—C5—C27—N4	−2.7 (2)	C27—N4—C31—C30	−0.9 (2)
C4—C5—C27—C28	177.36 (14)	C27—C28—C29—C30	−0.3 (2)
C5—N1—C1—C2	0.7 (2)	C28—C29—C30—C31	0.1 (2)
C5—N1—C1—C6	−179.79 (13)	C29—C30—C31—N4	0.5 (2)
C5—C27—C28—C29	179.78 (14)	C31—N4—C27—C5	−179.19 (13)
C6—N2—C10—C9	0.3 (2)	C31—N4—C27—C28	0.7 (2)
C6—C1—C2—C3	179.82 (13)		

### Hydrogen-bond geometry (Å, º)

Cg is the centroid of the C11–C16 ring.

**Table:**

*D*—H···*A*	*D*—H	H···*A*	*D*···*A*	*D*—H···*A*
C2—H2···N2	0.95	2.50	2.815 (2)	99
C4—H4···N4	0.95	2.46	2.778 (2)	100
C8—H8···O1^i^	0.95	2.59	3.529 (2)	170
C16—H16···*Cg*^ii^	0.95	2.81	3.669 (2)	151
C22—H22···O1^iii^	0.95	2.55	3.485 (2)	170

Symmetry codes: (i) *x*−1, *y*, *z*+1; (ii) *x*, −*y*+1/2, *z*+1/2; (iii) *x*, *y*, *z*+1.
